# Interplay of disability, caregiver impact, and out-of-pocket expenditures in Duchenne muscular dystrophy: a cohort study

**DOI:** 10.1186/s41687-022-00425-2

**Published:** 2022-03-10

**Authors:** Carolyn E. Schwartz, Roland B. Stark, Katrina Borowiec, Ivana F. Audhya, Katherine L. Gooch

**Affiliations:** 1 DeltaQuest Foundation, Inc., 31 Mitchell Road, Concord, MA 01742 USA; 2 Departments of Medicine and Orthopaedic Surgery, Tufts University Medical School, Boston, MA USA; 3 Department of Measurement, Evaluation, Statistics, and Assessment, Boston College Lynch School of Education and Human Development, Chestnut Hill, MA USA; 4 Sarepta Therapeutics, Cambridge, MA USA

**Keywords:** Muscular dystrophy, Duchenne, Caregivers, Quality of life, Disability, Out of pocket, Health expenditures

## Abstract

**Background:**

Providing caregiving support to people with Duchenne muscular dystrophy (DMD) is challenging, beginning in early childhood, and continuing through the progression of multidimensional disability. This study addressed the interplay between caregiver impact, out-of-pocket expenditures, and DMD disability. To examine these interconnections, we investigated the association between caregiver impact domains and out-of-pocket expenditures; and the presence of clusters in caregivers on the basis of DMD-related disability domains in the patients for whom they provided caregiving support.

**Methods:**

This web-based study recruited 566 DMD caregivers (140 males, 426 females; mean age 41.6 years, SD 8.8, range 21–72), examining caregiver impact using the DMD Caregiver Impact Measure, PROMIS-derived parent-proxy (PPP) measures of their child’s disability, and items tapping out-of-pocket expenditures related to home and vehicle accommodations and assistive devices. T-tests compared caregiver impact scores by out-of-pocket expenditures incurred. Latent Profile Analyses (LPA) were conducted to generate impact profiles related to child’s disability as reported by caregiver proxies.

**Results:**

Higher out-of-pocket expenditures were generally associated with worse impact on the subscales, but several expenditures (e.g., kitchen, bathroom, scooter) were associated with lower impact. LPA indicated that the four-group solution provided the best relative fit and yielded good profile separation (entropy = 0.91). Caregivers with lowest impact reported the highest mobility, cognitive, and upper extremity functioning of their DMD care recipients, whereas the highest caregiver impact was driven by their care recipient’s negative affect and fatigue. The upper-middle impact group showed great variability in proxy-disability domains, whereas the lower-middle group had similar levels of disability across domains. Profiles were represented across all child ages.

**Conclusion:**

Out-of-pocket expenditures were often associated with worse caregiver impact, but some associated with milder impact (i.e., bathroom or kitchen modification, investing in a ceiling lift or medical scooter). While their son’s level of disability and age were related to impact on the DMD caregiver, the domains giving rise to highest caregiver impact were not the most visible aspects of disability, such as mobility, but rather negative affect and fatigue. Other contextual attributes are likely implicated, and will be addressed in the companion paper.

**Supplementary Information:**

The online version contains supplementary material available at 10.1186/s41687-022-00425-2.

## Introduction

Providing caregiving support to chronically-ill children is challenging, particularly in the context of a diagnosis and disease onset in the early-childhood and a progressive disease trajectory. The long-term effects of caregiving are well-documented, and can include poorer health, quality of life (QOL), and well-being [1–5]. Treatment effectiveness is relevant to caregiver impact, since better management of the child’s disease can reduce the stressful vicissitudes of life [6]. Not all caregivers suffer equally, so understanding the personal factors associated with better and worse outcomes can be useful for designing appropriate and targeted interventions.

A particularly challenging diagnosis for caregiving is Duchenne muscular dystrophy (DMD). DMD is a progressive, rare, and irreversible neuromuscular disorder occurring primarily in males—1 in 5050 live births [7–9]. Usually diagnosed by age 5, the disorder presents as delayed development that includes motor difficulties [10] and may include cognitive impairment and attention deficit disorders [11]. On average by age 10–12, progressive muscle weakness leads to loss of ambulation, upper-limb function problems, and comorbid conditions such as scoliosis and muscular contractures [10]. By age 15, patients experience increased difficulty breathing and life-threatening heart and lung conditions [12]. DMD patients face profound uncertainty regarding lifespan, typically dying in their 20 s to early 30s^12^, although medical advances [7] have led to longer life expectancies [13].

Research on DMD caregiving suggests that impact increases with disease progression [14] and that caregiving demand is associated with worse QOL outcomes [15–20], sleep health [21], and lower work productivity [22]. Cognitive and mental-health problems in the DMD patient are associated with substantially worse financial burden for caregivers [23]. While much of this research has relied on small sample sizes without a comparison group [20], recent work has responded to these deficiencies by synthesizing findings from empirical work [20, 24]. Other recent work based on a larger sample and which included a nationally representative sample found that DMD caregivers reported worse QOL and faced more hurdles in their educational and work life, achieving less than one would expect given their education level [25]. Impact was most problematic during their child’s teenage years, a period associated with disability progression in domains directly relevant to independence [25].

The present study sought to address how best to characterize the interplay between caregiver impact, DMD disability, and DMD-necessitated out-of-pocket expenditures. We addressed questions such as “How do out-of-pocket expenditures link with caregiver-impact domains?” and “How do caregiver-impact domain levels and DMD disability domain levels cluster together?” By describing these interconnections, we hope to better understand the impact of DMD on caregivers. To examine these interconnections, we investigated the association between caregiver-impact domains and out-of-pocket expenditures; and the presence of clusters in caregivers on the basis of DMD-related disability domains in the patients for whom they provided caregiving support.

## Methods

### Sample and procedure

This study recruited participants via Rare Patient Voice, LLC; patient-advocacy groups; and word of mouth (i.e., snowball technique). Eligible participants were age 18 or older, able to complete an online questionnaire, and were providing caregiving support to a family-member with DMD at least two years old, usually their son. Ineligibility criteria included not being of age 18, not being able to complete an online questionnaire, not providing caregiving support to a family-member with DMD who was at least two years old, and not being able to provide informed consent. Caregiver-participants with motor, visual, and/or other problems that made it difficult for them to complete the web-based survey instrument, enlisted the assistance of a household member to enter their survey answers. This survey was administered through the HIPAA-compliant, secure Alchemer engine (www.alchemer.com) from June to November 2020. Dillman’s Tailored Design Method [26] was followed to maximize response and data quality. This method specifies a process regarding all communication to study participants (e.g., structure and content of all emails) and survey implementation to reflect professionalism and attention to detail (e.g., end-user experience of survey, pretesting, tracking responses and persevering in email invitations, etc.).

Recruitment was stratified by age of the caregiver’s child with DMD: 2–7, 8–12, 13–17, and >  = 18. These strata broadly correspond to the disease-related phases of progression: ambulatory (age 2–7), transitional (up to age 12), and non-ambulatory (age >  = 13), with increasing dependence and involvement of other systems as the person ages into adulthood (age >  = 18). If caregivers had more than one person with DMD for whom they were providing caregiving support, they were asked to report on the eldest or most disabled person (the index patient). Caregivers were paid $75 honoraria for their time completing the survey. The protocol was reviewed and approved by the New England Independent Review Board (NEIRB #20201623), and all participants provided informed consent prior to beginning the survey.

### Measures

Person-reported outcomes (PROs) were used to characterize and describe caregiver-impact groups. Characterizing the groups focused on caregiver impact, DMD care recipient’s disability, and out-of-pocket expenditures.

*Caregiver Impact* was assessed using a DMD adaptation of the Hemophilia Caregiver Impact measure [27, 28]. Items were modified to refer to DMD and were pretested for content via telephone interviews. Evidence for the content validity, construct validity, and internal consistency reliability of this DMD adaptation of the measure were obtained through initial analyses of this data set [25]. The resulting 39-item DMD Caregiver Impact measure (DCI) includes seven negative-impact subscales (Practical, Physical, Financial, Symptom, Lifestyle, Social, and Emotional) and one positive-impact subscale (Positive Emotions). Scores use a standardized T-score metric, with a mean of 50 and a standard deviation of 10.

*Care recipient’s disability* was measured using the Patient-Reported Outcome Measurement Information System (PROMIS) Parent Proxy (PPP) item banks [29]. These were adapted for use with and validated in this same sample of DMD caregivers in an earlier study [30]. The PPP measure included four domain scores where higher scores reflect better outcomes (mobility, upper extremity, cognitive function, and positive emotion) and four where higher scores reflect worse outcomes (fatigue impact, strength impact, negative affect, and sleep device symptoms). In the present work, all scores used a standardized T-score metric, with a mean of 50 and a standard deviation of 10.

*Out-of-pocket expenditures* assessed included binary variables reflecting the following 11 home and vehicle modifications that might have been implemented to accommodate their child’s DMD: entrance, bathroom, doorways, van, bedroom, kitchen, elevator, new home, ceiling lift, scooter, and other (open text option to specify). We refer to these modifications as “expenditures” because they involve financial costs, in contrast to other accommodations that might not be directly related to financial costs, such as choosing clothing items that do not involve buttons.

### Statistical analysis

Initial analyses sought to use Rasch modeling to summarize the out-of-pocket expenditures on a single unidimensional scale, but these analyses did not result in good item- or person-fit or strong correlations with the DCI subscales. Accordingly, we used a simpler approach, computing independent-sample t-tests and related effect sizes for group differences on each out-of-pocket category and DCI subscale. Using Cohen’s criteria, a *d* of 0.2 to 0.49 is considered a small effect size, 0.5 to 0.79 is medium, and 0.8 or greater is large[31].

To investigate the interplay of DMD caregiver impact and patient disability domains, two multivariate analyses were implemented and compared. Hierarchical cluster analysis [32] was used because it accommodates binary, categorical, and continuous variables and skewed distributions. It is flexible and effective even in small samples. Three- and four-cluster solutions were examined, and a four-cluster solution was selected because it better discriminated groups, had a reasonable sample size within each cluster, had large between-cluster variance relative to within-cluster variance (i.e., eta-squared in analysis of variance (ANOVA)), and because multinomial logistic regression showed good ability of the 16 DCI and PPP scores to predict cluster membership. A radar chart was used to display the clusters by DCI and PPP scores.

The second multivariate approach utilized Latent Profile Analysis (LPA) [33]. LPA is a latent variable model based, like factor analysis and modeling, on item response theory (IRT) [33]. The latent variable is conceptualized as continuous in factor analysis and IRT, but as categorical in Latent Class Analysis (LCA) and LPA. While LCA uses categorical indicators to measure the latent trait, LPA uses continuous indicators. LPA models were tested evaluating one-, two-, three-, and four-profile solutions. Final model selection was based on entropy values (criterion of > 0.80), an index based on the uncertainty of classification [34] that indicates the level of separation between classes [35, 36]; p-values of the Lo-Mendell-Rubin (LMR) [37] and bootstrap likelihood ratio (BLRT) [38, 39] tests; and the Akaike and Bayesian Information Criteria (AIC and BIC) [40–42], both of which are based on the maximum likelihood estimates of the model parameters for selecting the most parsimonious and informative model [35]. Cross-tabulations were obtained to examine profiles by child age.^1^ The two multivariate approaches were compared on the basis of average explained variance of the 16 included scores, and on the strength of the association between membership categories (Cramer’s V).

#### Statistical considerations and power related to sample recruitment

We aimed to recruit a minimum of 130 caregivers per age stratum, allowing for 20% attrition while still yielding 80% power, α = 0.05, to detect a medium effect size in a multiple regression model with seven covariates [32]. A medium effect size is a common standard for clinical significance [43].

IBM SPSS version 27 [44] and Mplus version 8.5 [36] were used for all analyses.

## Results

### Sample

Table 1 provides descriptive information on the 566 caregivers in the study sample. Because honoraria were only paid for complete surveys, the vast majority of surveys received were completed fully (566 out of 576 or 98.3%) and missing data was not an issue. The sample was initially recruited via Rare Patient Voice, LLC (9%) and patient-advocacy groups (15%), with the remaining 76% recruited through word of mouth. The sample had a mean age of 41.6, and 75% were female. Participants were drawn from the contiguous United States, and 92% were white, 8% black, and 10% Hispanic. Most respondents were married or in a domestic partnership; over half were employed; and the median level of education was a two-year university degree. Caregivers had an average of 1.3 comorbid health conditions out of 15 presented, with the most prevalent being back pain, depression, insomnia, and arthritis. Most were never-smokers, and the average body mass index reflected being overweight.

**Table 1:** Descriptive statistics of caregivers (n = 566)

Variable	Mean	SD	Min	Max
Age	41.6	8.8	21	72
	**Frequency**	**%**		
Gender				
Male	140	25%		
Female	426	75%		
US region				
South Atlantic	103	18%		
East North Central	70	12%		
Pacific	91	16%		
Middle Atlantic	63	11%		
East South Central	68	12%		
West South Central	45	8%		
Mountain	30	5%		
West North Central	28	5%		
New England	21	4%		
Non-contiguous	1	0%		
Missing	46	8%		
Marital status				
Never married	31	5%		
Married	453	80%		
Cohabitation/domestic partner	40	7%		
Separated	11	2%		
Divorced	23	4%		
Widowed	5	1%		
Missing	3	1%		
Race (check all that apply)				
Black	47	8%		
White	519	92%		
Other	21	4%		
Hispanic ethnicity				
Yes	57	10%		
No	490	87%		
Missing	19	3%		
Level of education				
Less than 12th grade	6	1%		
High school diploma	59	10%		
Technical (vocational) degree	66	12%		
Some college	92	16%		
2-year university degree	89	16%		
4-year university degree	171	30%		
Masters degree	53	9%		
Doctoral degree	5	1%		
Missing	25	4%		
Recruitment source				
Rare patient voice	49	9%		
Patient advocacy groups	87	15%		
Word of mouth	428	76%		
Missing	2	0%		
Comorbidities				
Comorbidities, out of 15 presented specific comorbidities^a^	1.3	1.7	0	9
	**Frequency**	**%**		
Arthritis	76	13%		
Asthma	50	9%		
Back pain	189	33%		
Cancer now or in the past	18	3%		
Depression	131	23%		
Diabetes	20	4%		
Heart disease	11	2%		
High blood pressure	53	9%		
Insomnia	117	21%		
Kidney disease	3	1%		
Liver disease	5	1%		
Lung disease	3	1%		
Stroke	3	1%		
Ulcer or stomach disease	18	3%		
Other	66	12%		
	**Mean**	**SD**	**Min**	**Max**
Body mass index	26.9	6.0	16.5	40.0
Smoking status	**Frequency**	**%**		
Never smoked	447	79%		
Used to smoke	58	10%		
Some days currently	23	4%		
Every day currently	35	6%		
Missing	3	1%		
Work status				
Employed	323	57%		
Unemployed	202	36%		
Retired	10	2%		
Disabled due to medical condition	10	2%		
Missing	21	4%		
Hours worked per week				
Does not apply	242	43%		
< 20	15	3%		
20–29	41	7%		
30–39	75	13%		
40+	193	34%		

^a^A non-response was counted as the absence of the comorbidity in question

### Caregiving context

Caregivers reported providing support to one to five people with DMD (mean = 1.1, SD = 0.4) (Table 2), representing equally four age strata: age 2–7, 8–12, 13–17, and 18 or older. Caregivers reported having up to eight children, up to three of whom had DMD. Families had an average of two people other than the caregiver providing this support. Caregivers were almost all (97%) parents of the DMD index person (Table 2). All index DMD care recipients were male, as expected. They had a mean age of 13.5 and had an average of 1.6 comorbidities among the separate list of 11 presented, the most prevalent of which were anxiety, learning disabilities, attention-deficit, scoliosis, sleep disorder, overweight, and depression.

**Table 2:** Descriptive statistics of care-recipients (n = 566)

Variable	%			
Index child: % male		100%		
	**Mean**	**SD**	**Min**	**Max**
Age	13.5	6.7	2	42
	**Frequency**	**%**		
Age 2–7	133	23%		
Age 8–12	131	23%		
Age 13–17	133	23%		
Age 18+	169	30%		
Years cared for by this caregiver	11.6	7.0	0	42
Number of people with DMD caring for	1.1	0.4	1	5
Total number of children	1.9	1.0	0	8
Number of children with DMD	1.1	0.3	0	3
Number of supports living in the home	2.1	0.8	0	3
Caregiver's relationship to DMD index person	**Frequency**	**%**		
Parent	549	97%		
Sibling	3	1%		
Other relative	9	2%		
Paid caregiver	0	0%		
Other	5	1%		
	**Mean**	**SD**	**Min**	**Max**
Comorbidities, out of 11 presented specific comorbidities^a^	1.6	1.8	0	9
	**Frequency**	**%**		
Anxiety	204	36%		
Asthma	45	8%		
Attention deficit	91	16%		
Autism spectrum disorder	45	8%		
Depression	74	13%		
Diabetes	11	2%		
Epilepsy	17	3%		
Overweight	96	17%		
Learning disabilities	130	23%		
Scoliosis	85	15%		
Sleep disorder	96	17%		

^a^A non-response was counted as the absence of the comorbidity in question

Table 3 provides descriptive statistics on the measures used to create caregiver-impact profiles: the DMD Caregiver Impact, the PROMIS Parent Proxy measures, and the out-of-pocket expenditures. Additional file 1: Table S1 provides the descriptive statistics on the raw scores of the DMD Caregiver Impact measure. In terms of out-of-pocket expenditures, more than half of the sample reported modifying the home entrance or bathroom. The other prevalent expenditures were modifying the inside doorways, purchasing a handicap-accessible van, and modifying the care recipient’s bedroom, all reported by at least 43%.

**Table 3:** Descriptive statistics of measures used to create caregiver-burden groups (N = 566)

Construct	Measure		DMD caregiver			
			Mean	SD	Min	Max
DMD caregiver impact	Practical impact		50	10	36	81
	Symptom impact		50	10	24	67
	Lifestyle impact		50	10	28	74
	Social impact		50	10	28	73
	Physical impact		50	10	34	77
	Emotional impact		50	10	33	74
	Financial impact		50	10	32	74
	Positive emotions^a^		50	10	15	64
PROMIS parent proxy measures of DMD child's disability	Fatigue impact		50.6	9.5	34	73
	Strength impact		50.5	9.5	35	68
	Negative affect		50.0	9.6	32	76
	Sleep-device symptoms		8.8	3.7	4	18
	Cognitive function^a^		50.5	9.4	22	63
	Upper extremity function^a^		50.3	9.6	34	66
	Positive emotions^a^		49.9	9.2	21	68
	Mobility^a^		31.9	13.6	13	65
Out-of-pocket costs			**Mean**	**SD**	**Min**	**Max**
	Count of expenditures		3.1	2.2	0	8
			**Frequency**	**%**		
		Modified home entrance	380	67%		
		Modified bathroom	303	54%		
		Modified inside home doorways	278	49%		
		Purchased handicap-accessible van	259	46%		
		Modified bedroom	241	43%		
		Modified kitchen	144	25%		
		Installed elevator	81	14%		
		Moved to or build new home	26	5%		
		Installed ceiling lift	16	3%		
		Purchased scooter	12	2%		
		Other	4	1%		

^a^Higher scores indicate better functioning; otherwise higher indicates worse

### Relationship between caregiver impact scores and out-of-pocket expenditures

Table 4 shows the relationship between DMD Caregiver Impact subscales and out-of-pocket expenditures. Only Cohen’s *d* statistics of small or larger ES are shown. Results suggest that some out-of-pocket expenditures were related to multiple aspects of caregiver impact, whereas others showed no association across domains. Specifically, the expenditures linked with the highest negative impact were new home, ceiling lift, van, and entrance modifications. The expenditures that had notable associations with negative impact, albeit on fewer domains, were bedroom and bathroom modifications and purchasing a medical scooter. Expenditures involving doorways and elevators were not associated with any DCI impact. Stated differently, out-of-pocket accommodations were associated with caregiver Physical impact for six categories; with Symptom impact for five; with Lifestyle, Social, Emotional, and Financial impact for four; and with Practical and Positive Emotions impact for two.

**Table 4:**
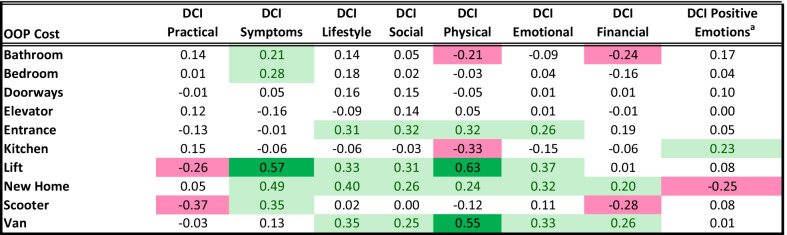
All caregiver impact score differences by out-of-pocket costs (Cohen's d)

Each Cohen's d statistic represents (mean for those making specified out-of-pocket accommodation—mean for others)/pooled SD. Values shown only when |d|> = 0.2

^a^Higher scores indicate better outcome

A second notable pattern shown in Table 4 is that in some cases the out-of-pocket expense was associated with *lower* caregiver impact. For example, kitchen modifications were associated *only* with better caregiver outcomes: lower physical impact and improved caregiver positive emotions. Bathroom modifications were associated with lower physical and financial impact, though with higher symptom impact. Ceiling lift was associated with a lower practical impact, despite higher symptom, lifestyle, social, physical, and emotional impacts. Medical scooter was associated with lower practical and financial impacts despite the higher symptom impact.

### Relationship between caregiver impact and DMD patient’s disability

Results of each multivariate analysis yielded a similar solution (4 groupings) with a strong association between groupings (Cramer’s V = 0.55). Each hierarchically-formed cluster had a reasonably large sample, and there was large between-cluster variance relative to within-cluster variance (11 out of 16 variables in ANOVA models had eta^2^ > 0.30). The four-cluster solution showed good discrimination between clusters on 2- and 3-D scatterplots, and multinomial logistic regression using cluster membership as the dependent variable showed good ability of the 16 characteristics (i.e., DCI and PPP scores) to predict cluster membership.

Results of the LPA supported two-, three- and four-profile solutions. We chose a four-profile solution rather than a three-profile solution because it had better separation between profiles (entropy = 0.91), a reasonably large sample within each profile, and good values for the other model-fit statistics (i.e., lowest BIC, lowest AIC, and non-significant LMR test result comparing the four- to three-profile solutions) (Additional file 1: Table S2). The four-class solution also seemed to distinguish middle groups best. Additional file 1: Table S2 shows model-fit statistics for all LPA models tested.

A comparison of the cluster and LPA solutions suggested that the latter explained more variance on average in the 16 DCI and PPP scores on which they were based (average eta^2^ = 0.33 vs. 0.37, respectively). The LPA also has the advantage of providing more formal metrics of model fit. We thus worked with the four LPA profiles for subsequent descriptive analyses.

**Fig. 1:**
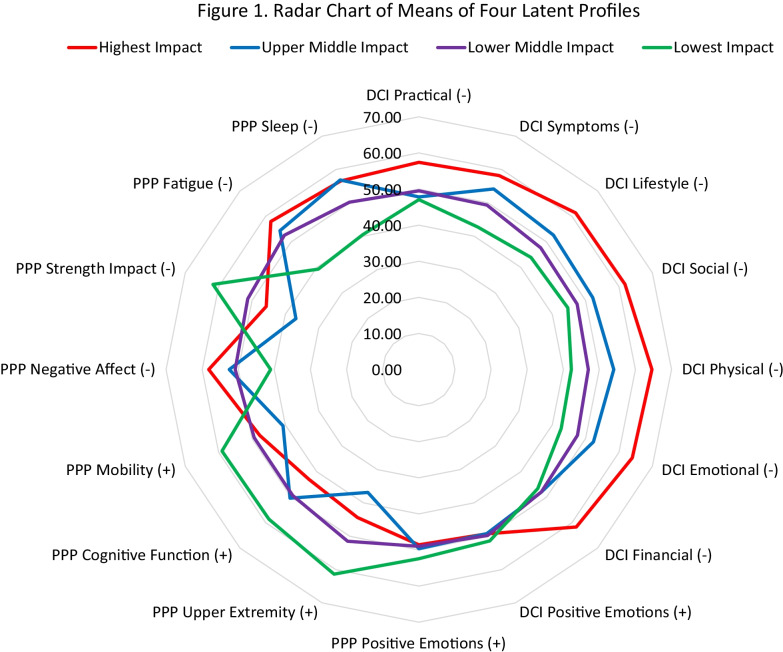
Radar chart of the DCI and PPP means by latent-profile membership. Higher mean scores are farther from center of the chart, and the scoring direction is shown in parentheses after each subscale name

Figure 1 shows a radar chart of the DCI and PPP mean scores by latent-profile membership. Higher mean scores are farther from center of the chart, and the scoring direction is shown in parentheses after each subscale name. The green line reflects the profile with the lowest caregiver impact. Their worst PPP score is in the domain of strength impact, and their DMD care recipients have the highest mobility, cognitive, and upper extremity functioning proxy scores. The red line reflects the profile with the highest caregiver impact, with highest scores on all DCI impact subscales. These caregivers’ DMD care recipients have the worst PPP negative affect and fatigue scores. In between are the purple and blue lines summarizing the remaining two profiles. The purple-profile members have lower levels of impact than the blue, and their DMD care recipients have consistent, moderate levels of disability across all PPP domains. The blue profile members have higher levels of impact than the purple (and green), and their DMD care recipients’ disability is quite variable: poor upper extremity functioning, mobility, and negative affect, but relatively high functioning on cognitive and strength impact PPP domains. Of note, the four profiles did not differ appreciably on DCI positive emotions or PPP Positive Emotions scores.

**Fig. 2:**
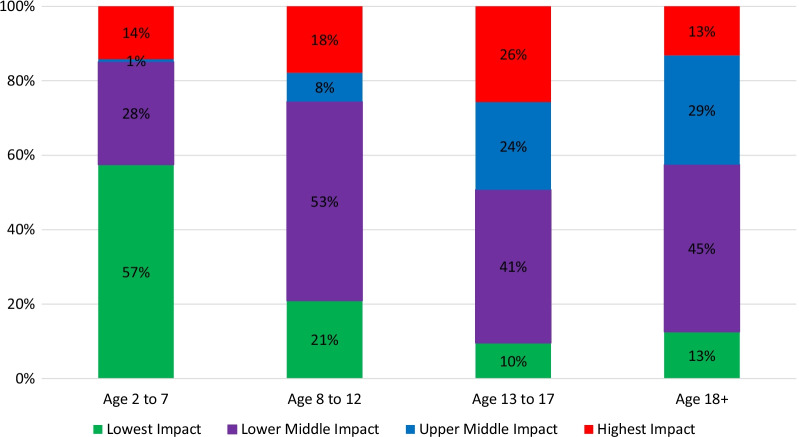
Stacked bar chart of most likely latent profile by DMD care recipient age

Figure 2 shows a stacked bar chart of most likely latent profile membership by DMD Care Recipient Age category. As expected by the natural history of disease progression, the lowest impact profile was less likely to occur among caregivers of DMD people in late childhood (age 13–17) and adulthood. Counter to expectation, the highest impact profile was similarly likely among caregivers of people in early childhood and adulthood, and most likely among caregivers of teenagers. The upper middle impact profile grew increasingly likely with older age of the DMD care recipients, whereas the lower-middle impact profile was most prominent in middle childhood care recipients, diminishing slightly as the care recipient aged. Although the upper-middle profile was very unlikely at the lowest age (1%), all impact profiles were present at all ages of the care recipient. This suggests that factors other than the care recipient’s disability influence the experience of caregiver impact.

## Discussion

Providing DMD caregiving support means a lifetime of challenges, beginning when a son is diagnosed in early childhood through the complex and difficult vicissitudes of disability progression over late childhood, adolescence, and adulthood. Although one might expect that caregiver impact would grow increasingly prominent as one’s son ages, the higher-impact groups were represented across the full age distribution of DMD care recipients. Of note, the PPP scores giving rise to highest caregiver impact were not the most visible aspects of disability, such as mobility, but rather negative affect and fatigue.

Out-of-pocket expenditures were often associated with worse caregiver impact, particularly for symptom, lifestyle, social and emotional impacts. In contrast, some out-of-pocket expenditures were associated with an attenuation of caregiver impact; these included modifying a bathroom or kitchen and investing in a ceiling lift or medical scooter. Thus, accommodations to the home in response to a child’s growing disability may help some aspects of impact despite the physical, social, emotional, and financial impacts of these expenditures. Using Verbrugge and Jette’s concept of the disablement process, such expenditures may reduce the patient’s gap between personal capability and environmental demand, and attenuate the impact of DMD on the caregiver [45].

It was notable that the four impact groups did not differ with regard either to the caregivers’ or care recipients’ levels of positive emotions (shown in Fig. 1). One might expect that caregiver impact would be associated with one or both indicators of well-being. This finding is, however, consistent with our finding on the same sample that DMD caregivers did not differ from the comparison participants in resilience [25]. It is also consistent with recent work done with hemophilia caregivers suggesting that caregivers who make the best of a difficult situation tend to adopt cognitive habits characterized by both acknowledging and accepting the limitations imposed by caregiving while at the same time adopting positive-focused approaches to thinking about their caregiving experience (e.g., an awareness of the personal growth enabled by caregiving) [46]. Such a balance enables an “alchemy” of the caregiving experience, perhaps by aiding the individual to reframe challenges, possibly by increasing a ‘positive balance’ in their QOL [47]. Thus, these similar scores across impact groups on positive emotions may provide a key to understanding how DMD caregivers manage to maintain the strength to persevere despite their many challenges.

The present study has a number of strengths, including its large sample size, comprehensive measurement of relevant constructs, application of multivariate methods, and its reliance on ES rather than *p*-values to ensure the clinical relevance of our findings. Its limitations must, however, be acknowledged. First and foremost, the data are cross-sectional in nature and so strict causal statements cannot be made. For this reason, we have focused throughout on describing findings in terms of “association” rather than causal effect. Second, the sample over-represents females, an issue that often arises with caregiver research [1, 3, 48, 49]. Third, the sample under-represents non-White races and Hispanic ethnicity. Fourth, our definition of out-of-pocket expenditures could be interpreted as out-of-pocket expenditures or economic burden as we have defined, but could also indicate a degree of adaptation that both care recipient and caregiver have responded to the care needs. Finally, we did not collect information on health insurance coverage. Future research might over-sample non-White and Hispanic DMD caregivers to enable description of their experiences using the same comprehensive measurement strategy and adding the collection of health insurance coverage.

In summary, this study found that DMD caregivers’ experience of impact varies widely in response not only to their son’s domain(s) of disability and age, but also as a function of the necessary accommodations and assistive devices to which they have access. In our companion paper [50], we examine other constructs to yield a more comprehensive ‘story’.

## Supplementary Information

**Additional file 1. Table S1**: DMD caregiver impact measure descriptive statistics. **Table S2**: Summary of latent profile analysis model fit results.

## Data Availability

The study data are confidential and thus not able to be shared.

## Abbreviations

ADL: Activities of daily living
AIC: Akaike’s Information Criterion
ANOVA: Analysis of variance
BIC: Bayesian Information Criterion
BLRT: Bootstrap likelihood ratio
COVID: Novel coronavirus-2019 pandemic
DCI: DMD Caregiver Impact measure
DMD: Duchenne muscular dystrophy
ES: Effect size
IRT: Item response theory
LMR: Lo–Mendell–Rubin
LPA: Latent profile analysis
NIH: National Institutes of Health
PRO: Person-reported outcome
PROMIS: Patient-Reported Outcome Measurement Information System
PPP: PROMIS Parent Proxy
QOL: Quality of life
SD: Standard deviation

## Footnotes

1 Further, with a sample size of 566, the number of people per cell would be too small to obtain a robust solution using a multigroup model in Mplus (4 classes × 4 groups × 16 indicators is likely too much for our data set of 566).
