# Supplementary material for: Interplay of disability, caregiver impact, and out-of-pocket expenditures in Duchenne muscular dystrophy: a cohort study
Source: J Patient Rep Outcomes. 2022 Mar 10;6:21. doi: 10.1186/s41687-022-00425-2 (PMC8908951; doi:10.1186/s41687-022-00425-2)
Supplement: Supplementary file 1 — Additional file 1. Table S1: DMD caregiver impact measure descriptive statistics. Table S2: Summary of latent profile analysis model fit results. [file 41687_2022_425_MOESM1_ESM.pdf]

Supplementary Table 1. DMD Caregiver Impact Measure Descriptive Statistics

DMD Caregiver Impact (DCI) Subscale	Raw Mean	Raw SD	Raw Min	Raw Max	Cronbach's Alpha
Practical Impact	2.25	0.89	1	5	0.88
Symptom Impact	3.42	0.92	1	5	0.88
Lifestyle Impact	2.94	0.87	1	5	0.77
Social Impact	2.97	0.88	1	5	0.87
Physical Impact	2.51	0.93	1	5	0.89
Emotional Impact	2.67	0.96	1	5	0.90
Financial Impact	2.72	0.95	1	5	0.88
Positive Emotions	3.84	0.82	1	5	0.85

Supplementary Table 2. Summary of Latent Profile Analysis Model Fit Results										
Model (classes)	Log likelihood	AIC	BIC	Sample adjusted BIC	Entropy	Smallest class % (Most likely class)	LMR	BLRT	Most likely latent profile membership in 4-profile model	
							p-value	p-value	Count	Percentage
1	-33500.33	67064.66	67203.49	67101.91					237	41.87%
2	-32425.08	64948.16	65160.75	65005.2	0.913	46.64%	p<.001	p<.001	138	24.38%
3	-32017.85	64167.7	64454.05	64244.53	0.884	24.74%	p=.0192	p<.001	99	17.49%
4	-31856.48	63878.95	64239.05	63975.57	0.909	16.25%	p=.5561	p<.001	92	16.25%
5	*Problems with model identification*									

Note: Entropy values closer to 1 are better and above 0.8 is very good.
